# Circulating Tumor Cells in Breast Cancer Patients: An Evolving Role in Patient Prognosis and Disease Progression

**DOI:** 10.4061/2011/621090

**Published:** 2011-01-03

**Authors:** Holly Graves, Brian J. Czerniecki

**Affiliations:** ^1^Harrison Department of Surgical Research, Hospital of the University of Pennsylvania, 3400 Spruce Street, Philadelphia, PA 19104, USA; ^2^Rena Rowan Breast Center of the Abramson Cancer Center and the Department of Surgery, University of Pennsylvania, 3 Perelman, 3400 Civic Center Drive, Philadelphia, PA 19104, USA

## Abstract

In this paper, we examine the role of circulating tumor cells (CTCs) in breast cancer. CTCs are tumor cells present in the peripheral blood. They are found in many different carcinomas but are not present in patients with benign disease. Recent advances in theories regarding metastasis support the role of early release of tumor cells in the neoplastic process. Furthermore, it has been found that phenotypic variation exists between the primary tumor and CTCs. Of particular interest is the incongruency found between primary tumor and CTC HER2 status in both metastatic and early breast cancer. Overall, CTCs have been shown to be a poor prognostic marker in metastatic breast cancer. CTCs in early breast cancer are not as well studied, however, several studies suggest that the presence of CTCs in early breast cancer may also suggest a poorer prognosis. Studies are currently underway looking at the use of CTC level monitoring in order to guide changes in therapy.

## 1. Introduction

Breast cancer is one of the most common cancers affecting women. It is estimated that one in eight women will develop an invasive breast cancer at some point during her lifetime. In 2010, according to the American Cancer Society, approximately 207, 090 new cases of invasive breast cancer will be diagnosed and 39, 840 women will die from metastatic disease. In this era of molecular medicine, novel approaches are needed in the management of breast cancer. In the last several decades, circulating tumor cells (CTCs) have emerged as a unique target for understanding disease progression, prognosis, and treatment in breast cancer pathogenesis.

CTCs are tumor cells present in the peripheral blood. They are found in many different carcinomas but are not present in patients with benign disease [1]. Much of the research involving CTCs stems from studies involving disseminated tumor cells (DTCs). DTCs are tumor cells present in the bone marrow. Briefly, several studies have shown that patients with DTCs at the time of diagnosis have larger tumors, higher histologic grade, and a higher incidence of lymph-node metastasis, distance metastasis, and cancer-related death versus those patients without DTCs [2, 3]. Furthermore, detection of DTCs after systemic treatment is associated with increased risk of recurrence and decreased disease-free survival as well as decreased breast cancer-specific survival [4, 5]. Though DTCs have been more thoroughly studied, there are several studies that have documented a correlation between the occurrence of DTCs and CTCs in both primary and metastatic breast cancer [6–10]. Since bone marrow sampling is cumbersome, difficult to reproduce, and morbid for patients, emphasis has been placed on advancing CTC research. This paper will address the current methodologies of CTC detection, the prognostic role of CTCs in both early and advanced breast cancer, and the implication of CTCs in disease progression, treatment, tumor biology, and further research.

## 2. Evidence for CTC in Early Metastasis

It was previously thought that metastasis occurred late in disease progression; however, evidence from CTCs/DTCs has shown that metastasis may be an early event. This is supported by the fact that CTCs/DTCs are found in patients with early breast cancer. A recent study by Husemann et al. with transgenic (HER2/PyMT) mice showed that dissemination of tumor cells can occur at a preinvasive stage of the primary tumor. They also found both in mice and early human breast cancer that the presence of CTCs/DTCs was independent of tumor size [11]. However, even though occult tumor dissemination may occur early, not all patients with detectable CTCs/DTCs will develop overt metastases. Meng et al. looked at 36 breast cancer patients 7 to 22 years after mastectomy and found that 36% had evidence of CTCs with no evidence of clinical disease [12]. Similarly, in a large pooled analysis by Braun et al., only half of DTC-positive breast cancer patients relapsed over a ten-year period [3]. These CTCs/DTCs may be in a state of dormancy and the exact mechanism of transition to overt metastases is unclear. Likely factors involved in this transition include host microenvironment, host immune response, and genetic changes in the tumor cell.

## 3. Phenotypic Variability between CTC and Primary Tumor

Several studies have found genotypic variation between primary tumor and CTCs/DTCs of particular interest is the incongruent HER2 status between primary tumor and CTCs/DTCs. A recent study utilizing the CellSearch System in metastatic breast cancer found that 29% of HER2-negative primary tumors had HER2-positive CTCs and 42% of HER2-positive primary tumors had HER2-negative CTCs [13]. Another study by Fehm et al. looked at serum HER2 and CTCs in initially HER2-negative or HER2-unknown metastatic breast cancer patients. Of the 77 patients, 23/77 patients were HER2 positive based on either CTC detection or peripheral blood ELISA. HER2 concordance between CTCs and serum HER2 was 71%. HER2 status of the metastatic tissue was assessed in ten of these patients in which 2/10 had discordance between primary tumor and metastatic site [14]. Similar discrepancies have been reported in other studies, mostly ranging from 7 to 40%, as well as intermetastatic site variability [14–19]. Discordant HER2 status between primary tumor and CTCs/DTCs has also been reported in early breast cancer [20–23].

There has been conflicting evidence for treatment based upon CTC/DTC HER2 status. In another study by Meng et al., 9/24 patients initially HER2 negative acquired HER2 gene amplification throughout their disease process. These patients were either far advanced or had undergone previous chemotherapy or radiation. Four of the patients were treated with trastuzumab leading to one complete response and two partial responses [24]. Another study looked at trastuzumab response in 30 breast cancer patients stages 1–4 who had already completed standard therapy. All 30 of these patients had CK-19 mRNA-positive circulating and/or disseminated tumors cells present. Though only 33% of the primary tumors were HER2 positive, 83% of the CTCs and/or DTCs were HER2 positive. After trastuzumab therapy, 28/30 (93%) patients showed no CK-19 mRNA signal [25]. Similar results were reported from the National Surgical Adjuvant Breast and Bowel Project (NSABP) protocol B-31 suggesting a potential benefit from trastuzumab to HER2-negative patients [26, 27]. However, a large randomized phase 3 trial looked at randomization of trastuzumab with paclitaxel to metastatic breast cancer patients with HER2-negative primary tumors. Trastuzumab did not affect overall survival, response rate, or time to progression in non-HER2-overexpressing tumors [27]. A large European multicenter study (www.detect-study.de) is currently underway looking at CTC HER2 expression in metastatic breast cancer patients with HER2-negative primary tumors. This trial will look at several different techniques for determining HER2 status. It will also look at HER2-positive CTC response to HER2-targeted therapy [28].

## 4. CTC Detection Methods

If CTCs are to be used as surrogates for DTCs, then accurate and reproducible techniques are needed for CTC quantification. This is especially important when considering that CTC concentration in peripheral blood can be as low as one per 10^5^–10^7^ cells [29]. CTC detection occurs in a two-step process, enrichment, and identification. Several techniques are available for CTC enrichment. The Ficoll and OncoQuick systems utilize a density gradient centrifugation. These systems lack specificity as they separate CTCs and mononuclear cells from red blood cells and granulocytes [29]. Furthermore, CTCs can migrate between layers and the layers themselves can lose their integrity. Between the two systems, Gertler et al. found the OncoQuick system to be superior due its ability to select out mononuclear cells [30]. The ISET (Isolation by Size of Epithelial Tumor Cells) technique implores a filter consisting of 8 *μ*m pores to separate CTCs from leukocytes [31]. However, small CTCs can be lost, and large leukocytes can be retained by the filters leading to poor sensitivity and specificity [29]. In general, these techniques have fallen out of favor, and most researchers use immunological techniques for enrichment.

Immunomagnetic systems target an antigen with an antibody that is coupled to a magnetic bead. They then isolate the antigen-antibody complex via exposure to a magnetic field. Enrichment can occur through either positive selection where the antibody is targeted against a CTC antigen (CKs, EpCAM, HER2) or negative selection where the antibody is targeted against a leukocyte antigen (CD 45 or 61). To date, the only FDA-approved system is CellSearch, an immunomagnetic system that uses anti-EpCAM and anti-CD45 antibodies. The main limitation is heterogeneity of CTCs to express EpCAM. Furthermore, EpCAM is downregulated in malignant cells though a process called epithelial to-mesenchymal transition. Despite this, the CellSearch System has a high reproducibility rate [1, 32]. 

Once enrichment is completed, characterization of CTCs is achieved through molecular or immunological techniques. RT-PCR methodologies target tumor-specific antigens. This technique was initially considered to be sensitive; however, other authors have found several limitations to this technique [33, 34]. These limitations included false positives due to illegitimate gene transcription, contamination by pseudogenes, and transcription of markers present on nonmalignant cells [35, 36]. Furthermore, false negatives may arise if CTCs do not express the gene of interest [29]. Lastly, RT-PCR relies on cellular lysis which precludes further CTC analysis and assessment of CTC quantity. However, more recent developments in techniques allow for increased sensitivity and specificity by overcoming these pitfalls. Multimarker RT-PCR and novel primer designs avoid false positives [37]. In addition, advances in PCR technology with quantitative real-time RT-PCR (RT-qPCR) allows for cutoff values of transcripts between cancerous and noncancerous cells, thereby designating what values are tumor-cell derived [38, 39].

There are several immunological techniques used for the identification process. As described earlier, the CellSearch System uses immunomagnetic technology for enrichment. In the identification stage, these cells are fluorescently stained for cytokeratins (CK8, 18, 19), common leukocyte antigen (CD 45), and a nuclear dye (4,6-diamino-2-phenylindole (DAPI)). A fluorescent microscope then detects and identifies CTCs as those cells that are CK+/CD45−/DAPI+. Fiber-optic array scanning technology (FAST) also utilizes fluorescent anticytokeratin antibodies as well as DAPI counterstain. Stained cells are then exposed to laser-printing optics that excite 300,000 cells/second. This affords similar sensitivity and specificity to conventional automated digital microscopy with a 500-fold increase in speed [40]. Laser Scanning Cytometry (LSC) rapidly scans and relocates multimarker immune-labeled cells for visual examination to separate viable from nonviable cells [41]. 

Multiparameter flow cytometry has been utilized by several authors for the detection of CTCs since multiple surfaces markers and DNA ploidy can be evaluated [42–44]. As such, flow cytometry affords a high specificity and in one study demonstrated a higher specificity than RT-PCR [45]. More recently, microchip technology has been described for detection of CTCs. The “CTC-chip” uses a microfluid platform by which CTCs in whole blood samples that interact with microposts coated in anti-EpCAM antibody [46]. The authors demonstrated a sensitivity of 99% in a cohort of patients with metastatic cancers. Epithelial immunospot (EPISPOT) is a technique based on the enzyme-linked immunospot assay. EPISPOT detects only viable tumor cells as evidence by their ability to secrete proteins (CK-19, MUC-1) in short-term cell cultures [47].

## 5. Metastatic Breast Cancer

Most of the literature thus far has examined CTCs in metastatic breast cancer. Cristofanilli et al. looked at 177 metastatic breast cancer patients in a multicenter prospective trial using the CellSearch System and found that the presence of CTCs before initiation of therapy was a predictor of both decreased progression-free survival as well as overall survival. Through stratification according to progression-free survival, a cutoff value of 5 CTCs per 7.5 ml of blood was used to distinguish patients with a favorable versus unfavorable prognosis [48]. Several subsequent studies found similar conclusions [49, 50], and further followup data revealed elevated CTC counts at any point during therapy was associated with decreased progression-free and overall survival [15, 51, 52]. The prognostic value of CTCs has been shown to be superior to tumor burden, disease phenotype, and current imaging methodologies [53, 54]. CTCs also allow for molecular profiling. Gradilone et al. looked at CTC chemoresistance profiles in metastatic breast cancer patients using RT-PCR to quantify the number of multidrug-resistance-related proteins (MRPs) expressed. Those patients with greater than two MRPs expressed per CTC had a shorter progression-free survival then those with more drug-sensitive CTC profiles [17]. The next step in CTC research is currently being undertaken by the Southwestern Oncology Group (SWOG: S0500) via a phase three randomized trial looking at changing therapy versus maintaining therapy in metastatic breast cancer patients who have elevated CTC levels after the first followup visit upon treatment initiation.

Lastly, it has been recently shown in a mouse model for metastatic breast cancer that CTCs can also colonize their tumor of origin. This work completed by Kim et al. has been referred to as “tumor self-seeding.” In their experimental model, the primary tumor was able to be seeded by separate tumor masses, metastatic lesions, and from direct inoculation. They found that the primary tumor secretes several cytokines that attract the CTCs, such as IL-6 and IL-8. In turn, once the CTCs have infiltrated the tumor, they secrete factors that influence the primary tumor microenvironment, including tumor growth, angiogenesis, and leukocyte recruitment. Thus, once further elucidated, the factors involved in CTC-primary tumor interactions allow for potential therapeutic targets [55].

## 6. Early Breast Cancer

There have been few studies regarding CTCs and early breast cancer. The reported CTC positivity rate has ranged from 9.4 to 48.6% [20, 22, 23, 56–67]. Several of these studies have tried to identify primary tumor characteristics that would predict the presence of CTCs. A recent study by Krishnamurthy et al. looked at DTCs and CTCs in stage 1 and 2 breast cancer patients and found that the presence of both DTCs and CTCs was independent of lymph node status, tumor grade, tumor size, and receptor status [64]. This is in contrast with early findings of the SUCCESS trial. This trial is looking at CTCs at the time of primary diagnosis as well as during adjuvant chemotherapy. They report a positive correlation between lymph nodes status and CTCs [68]. Lang et al. looked at both CTCs and DTCs and found that only HER2 status of the primary tumor was associated with the presence of CTCs [62]. In contrast to previous studies, both Krishnamurthy et al. and Lang et al. did not find a correlation between the presence of CTCs with DTCs [62]. 

Several studies involving early breast cancer patients have shown that the presence of CTCs is associated with a worse prognosis. Wulfing et al. used a buoyant density gradient and immunomagnetic separation technique to look at HER2-positive CTCs in stage 1 through stage 3 breast cancer patients. They found that 17/35 (48.6%) patients had HER2-positive CTCs, of which twelve of these patients had HER2-negative primary tumors. The presence of HER2-positive CTCs was associated with a significantly decreased disease-free survival and overall survival [65]. This was validated by a recent large study of 216 patients using an RT-PCR technique to look at HER2-mRNA-positive CTCs [23]. 

Other studies have looked at RT-PCR techniques using mammaglobin A and CK-19. Ignatiadis et al. used a triple primer RT-PCR technique using CK19, mammaglobin A, and HER2 in 175 women with early breast cancer. They found that the presence of CK-19 mRNA-positive and mammaglobin A mRNA-positive CTCs prior to the initiation of adjuvant therapy was associated with a decreased disease-free survival [59]. However, a previous study by Ignatiadis et al. looking at 444 early stage breast cancer patients found that the presence of CK-19 mRNA-positive CTCs was associated with a reduced disease-free survival in only ER-negative, triple-negative, and HER2-positive patients [60]. Xenidis et al. looked at 167 node-negative breast cancer patients and found that the presence of CK-19 mRNA-positive CTCs was associated with both early clinical relapse and disease-related death [58]. Ntoulia et al. and Ferro et al. found that mammaglobin A mRNA positivity was associated with a poorer prognosis [61, 67].

## 7. Treatment Monitoring

One goal of CTC detection is to be correlate CTC levels to disease progression and response to treatment. In early breast cancer, some studies have found a correlation between initial CTC reduction upon therapy initiation and the final response of the tumor [69, 70]. However, most studies in early breast cancer do not support a correlation between CTC response and tumor response. The GeparQuattro study looked at CTC levels at the time of diagnosis and after neoadjuvant therapy in 213 large operable and locally advanced breast cancer patients. Neoadjuvant therapy included trastuzumab if the primary tumor was HER2 positive. The incidence of CTCs went from 21.6% before treatment to 10.6% after treatment. Fifteen percent of initially CTC-positive patients became CTC negative after treatment, and 8.3% of initially CTC-negative patients became CTC-positive after treatment. However, no significant correlation was found between CTC detection and the primary tumor's response to neoadjuvant therapy [20]. Pierga et al. found similar results in a study of 118 stage 2-3 breast cancer patients using the CellSearch System. Though 23/118 (19%) patients had a complete response to neoadjuvant therapy, changes in CTC count did not correlate with tumor response [66]. 

Though data is inconsistent regarding tumor response, most studies have found that the presence of CTCs does predict early relapse. The SUCCESS trial looked at 1,489 nonmetastatic breast cancer patients using the CellSearch System and found that pretreatment CTC detection was associated with reduced disease-free survival as well as overall survival, while post treatment CTC detection was only associated with reduced disease-free survival [68]. Similar results were shown in the previously described study by Pierga et al. [66]. The data from Pierga et al.'s study was further analyzed after a longer followup period (18 months versus 36.4 months) and concluded that preneoadjuvant CTC detection is a better predictor of overall survival and distant metastasis-free survival than post-treatment CTC detection [56]. Xenidis et al. looked at 437 early stage breast cancer patients and found both pre- and post-treatment CK-19 mRNA-positive CTCs to be associated with decreased disease-free and overall survival [58]. Apostolaki et al. looked at HER2 mRNA-positive CTCs in 214 stage 1 and 2 breast cancer patients. Initially HER2 mRNA positivity was 21%. Adjuvant chemotherapy was able to eliminate CTCs in 16/53 (30.2%) of patients. The presence of CTCs after treatment was associated with reduced disease-free interval [22]. Similar results were reported in a study looking at adjuvant therapy which found that an increase in CTC level of tenfold or higher, independent of an initial response, predicted early relapse [71]. 

Several studies have looked specifically at CTCs during treatment with hormonally therapy. Pachmann et al. recently found that escalating numbers of CTCs during Tamoxifen treatment was a strong predictor of relapse. This increase was also predictive of subsequent relapse during aromatase inhibitor treatment [72]. Furthermore, Xenidis et al. reported a reduced disease-free interval as well as overall survival with persistent CK-19 mRNA CTC positivity after treatment with Tamoxifen [73].

## 8. Conclusion and Future Directions

Evidence has shown that CTCs play a prognostic role in both early and metastatic breast cancer patients. In early breast cancer, the presence of CTCs allows clinicians to identify those patients that are at risk for recurrence and therefore may benefit from additional therapy. In both early and metastatic breast cancer patients, CTCs are an easily assessable source for monitoring treatment efficacy. Though results from the SWOG trial are pending, CTC monitoring may allow oncologists to change therapy earlier in disease progression. Lastly, with molecular and genetic characterization of CTCs, chemoresistance profiles should also be able to advise the clinician of the most efficacious chemotherapy regimens.

In terms of tumor biology, it is clear that circulating tumor cells are present in early breast cancer thus supporting the theory of early metastasis. One question yet to be answered is exactly how early in the neoplastic process does tumor dissemination occur. Studies have yet to look at the presence of CTCs in ductal carcinoma in situ (DCIS). Not all CTCs may lead to metastatic deposits as the metastatic niche may need to be created. Furthermore, even in early breast cancer, CTCs show great diversity compared to the primary tumor. Of particular interest is the diversity in HER2 status. It may be possible to target CTC/DTC to eradicate potential metastatic deposits. Targeting CTC using vaccines against HER2 and other pathways involved with breast cancer could theoretically decrease the probability of CTC seeding, recurrence, and/or metastasis [74–77].
